# Comment on: “Somatic CAG repeat instability in intermediate alleles of the HTT gene and its potential association with a clinical phenotype” by Ruiz de Sabando et al.

**DOI:** 10.1038/s41431-024-01587-x

**Published:** 2024-03-07

**Authors:** Magdalena Mroczek

**Affiliations:** 1 Department of Neurology, University Hospital Basel, University of Basel, Basel, Switzerland; 2 European Society of Human Genetics-Young Committee, Vienna, Austria

**Keywords:** Genetics research, Biomarkers, Translational research

The manuscript of Ruiz de Sabando et al. [1] focuses on the up-to-date topic of mechanisms of symptom development in the *HTT* IAs (intermediate alleles; 27–35 CAG repeats). The authors investigated the concept of somatic instability in blood, which has already been reported for full-penetrance alleles of HTT but has not yet been investigated in *HTT* IAs. The authors hypothesize that *HTT* IAs can trigger a mild, in particular neuropsychiatric Huntington disease (HD) phenotype, following the CAG-dependent somatic expansion [1]. The study was performed on the largest published cohort of symptomatic *HTT* individuals, including 191 IA carriers from the general population, identified in 11 HD centers in Spain and 164 HD family members from Hospital Universitario de Navarra (Spain). Although it could not be proved that *HTT* IAs somatic instability can trigger a mild HD phenotype, expansions in blood with short somatic gains may consist of one of the potential modifying factors contributing to symptoms development.

HD belongs to a group of 50 disorders caused by trinucleotide expansions in the DNA sequence. *HTT* alleles can traditionally be classified as normal, IAs, reduced-penetrance and full-penetrance, depending on the number of CAG repeats. Data derived from both the mathematical model [2] as well as from the human studies [3] accumulated substantial proof that repeat disease alleles can be tissue-specific and somatically unstable in full penetrance alleles leading to an affected individual being mosaic for multiple size alleles. Somatic instability was detected also in blood, although more pronounced in the brain and increased with age. In other expansion diseases, such as in premutation carriers in at the *FMR1* locus, a high level of somatic instability has been associated specifically with the presence of neuropsychiatric symptoms, such as attention deficit hyperactivity disorder (ADHD) [4].

By definition, intermediate alleles are not causative of HD and relatively common in the general Caucasian population (5–6%). Symptomatic IAs constitute only a small fraction of all IAs. Several recent studies confirmed that they may however be associated neurocognitive symptoms such as cognitive decline, depression, or apathy. In the study of Ruiz de Sabando et al. [1] 82 symptomatic *HTT* IA carriers presented with motor (85%), cognitive (27%) and/or behavioral (29%) signs. However, the age of symptom onset was not CAG somatic expansion dependent. The data from the total IAs cohort on the neurocognitive symptoms did not permit a thorough phenotypic analysis with only several individuals being sufficiently characterized. Four clinically well-characterized male IA carriers, members of three HD families, displayed irritability, verbal aggressiveness, perseverative thinking, apathy, deterioration of executive functions, memory problems and difficulty in learning new material. On the level of the total cohort investigated, a significantly higher frequency of IAs among symptomatic individuals in comparison to population controls could not be identified. Given that IAs in the general population occur with a 5–6% frequency, these individuals may also suffer from the coinciding other neurological disease.

Further, Ruiz de Sabando et al. [1] reported that instability of somatic expansion can be identified not only in HD patients but also in IAs. The somatic expansions in blood showed short somatic gains of +1 to +3 in reduced penetrance and +1 to +2 CAG repeats in IAs, respectively. No longer expansions were identified. This demonstrated that the number of repeats lies on the continuum rather than follows a strict pattern of current formal classification. However, here, similarly, as for the neurocognitive symptoms, the authors did not report an increase in the level of somatic expansion in blood DNA of symptomatic IA carriers versus population controls. It is to be mentioned that the somatic expansion shows a strong tissue specificity, with a high level of somatic expansions in the specific brain regions of HD patients in comparison to blood. However, brain samples are hardly available. The authors studied one brain of a 33 CAG carrier, from an HD family, presenting cognitive difficulties, irritability and mild motor alterations. The somatic expansion was found to have the highest ratio in the putamen (10.3%), and the lowest in the cerebellum (4.8%), which is in concordance with the data from the early HD patients. Also here expansions lied in a range of +1 to +2 CAG repeats and large-length expansions have not been detected.

Although the authors failed to demonstrate that CAG somatic expansion in *HTT* IAs can trigger the presence of the phenotype, they discuss the potential role of the short somatic expansions in blood as a potential modifying factor. Also, other genetic events identified in this cohort previously [5], such as an atypical 33 CAG IA/42 CAG allele in which the *CAA* repeat interruption was lost, may act as a potential genetic disease modifier.

Altogether the authors did not confirm that a low level of somatic expansion in *HTT* IAs can trigger the HD phenotype. However, the continuum of CAG expansions in blood with a range of short alleles in *HTT* IAs has been identified. Does it mean that somatic instability should no longer be considered a potential mechanism triggering the symptoms in IAs? Not necessarily. First of all, the somatic expansion follows a tissue-specific pattern so that particular brain regions would be more informative. Secondly, the phenotypic information available for the cohort reported is not sufficient to delineate a specific neuropsychiatric phenotype so larger longitudinal cohort studies are needed. It is probable that high CAG repeats in the IAs of the *HTT* gene may predispose to the development of different neurodegenerative disorders, sharing a common disease mechanism. Finally, the appearance of symptoms in IAs should be considered as a combination of different environmental and genetic modifying factors with a low level of CAG expression in blood being potentially one of them. The study of Ruiz de Sabando et al. [1] is a valuable starting point for a further discussion on the role of somatic instability as a modifying factor for developing especially neurocognitive phenotype in *HTT* IAs with practical implications for understanding disease mechanisms and impacting genetic counseling.

## References

[1] Ruiz de Sabando A, Ciosi M, Galbete A, Cumming SA, Spanish HD, Monckton DG et al. Somatic CAG repeat instability in intermediate alleles of the HTT gene and its potential association with a clinical phenotype. Eur J Hum Genet. 10.1038/s41431-024-01546-6. 10.1038/s41431-024-01546-6 PMC11220145 38433266

[2] Kaplan S, Itzkovitz S, Shapiro E (2007). A universal mechanism ties genotype to phenotype in trinucleotide diseases. PLoS Comput Biol.

[3] Kacher R, Lejeune FX, Noël S, Cazeneuve C, Brice A, Humbert S (2021). Propensity for somatic expansion increases over the course of life in Huntington disease. Elife.

[4] Aishworiya R, Hwang YH, Santos E, Hayward B, Usdin K, Durbin-Johnson B (2023). Clinical implications of somatic allele expansion in female FMR1 premutation carriers. Sci Rep.

[5] Ruiz de Sabando A, Urrutia Lafuente E, Galbete A, Ciosi M, García Amigot F, García Solaesa V (2023). Spanish HTT gene study reveals haplotype and allelic diversity with possible implications for germline expansion dynamics in Huntington disease. Hum Mol Genet.

